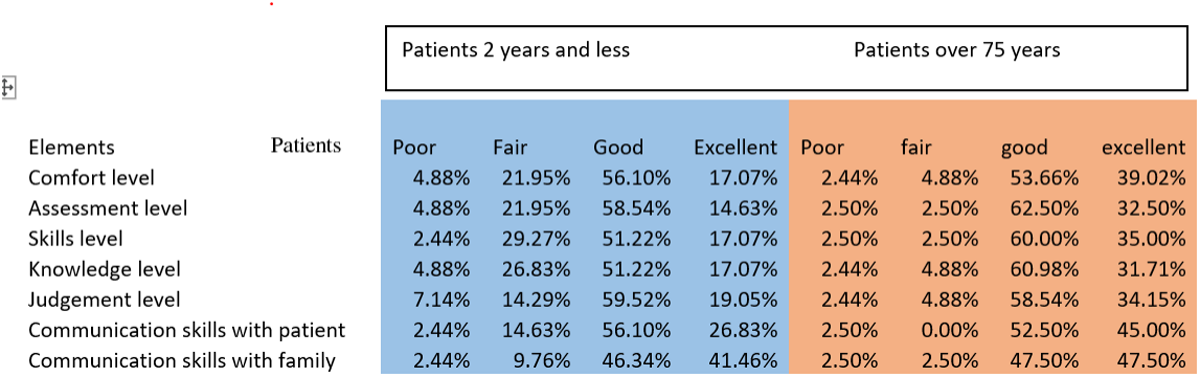# 745 Caring for Infants, Centenarians, and Everything in the Middle

**DOI:** 10.1093/jbcr/irad045.220

**Published:** 2023-05-15

**Authors:** Aemilio Ha, Frek Rita, Michelle Grywalski, Teresa Chin, Mira Bakas, Natalya Godes

**Affiliations:** UCI Health, Orange, California; UCI, Orange, California; UCI, Orange, California; UCI, Orange, California; UCI, Orange, California; UCI Health, Seal Beach, California; UCI Health, Orange, California; UCI, Orange, California; UCI, Orange, California; UCI, Orange, California; UCI, Orange, California; UCI Health, Seal Beach, California; UCI Health, Orange, California; UCI, Orange, California; UCI, Orange, California; UCI, Orange, California; UCI, Orange, California; UCI Health, Seal Beach, California; UCI Health, Orange, California; UCI, Orange, California; UCI, Orange, California; UCI, Orange, California; UCI, Orange, California; UCI Health, Seal Beach, California; UCI Health, Orange, California; UCI, Orange, California; UCI, Orange, California; UCI, Orange, California; UCI, Orange, California; UCI Health, Seal Beach, California; UCI Health, Orange, California; UCI, Orange, California; UCI, Orange, California; UCI, Orange, California; UCI, Orange, California; UCI Health, Seal Beach, California

## Abstract

**Introduction:**

Age specific considerations are integral part of quality care. Several burn units care for all age groups in a single unit, needing additional resources and infrastructure to provide quality care for extremes of age.

**Methods:**

An anonymous online survey was conducted among the multidisciplinary burn team members of a burn unit that cares for both pediatric and adults. Data for the last 4years found that 12-18% of all burn admissions were 2years or less, while 5-6% of burn admission were of over 75 years of age.

**Results:**

45 members responded to the survey, including 56 % RNs and the remaining included therapists, pharmacist, psychologist, social worker, child life specialist, and case manager. The results showed that team members felt more comfortable caring for geriatric patients than pediatrics. Staff members reported higher importance for having intubated pediatric patient with leverage of 1:1RN assignment as well as daily rounding by pediatric intensivist and pediatric pharmacist. Majority (73%) of respondent s reported good or excellent in their assessment, skill, knowledge, and communication ability for caring pediatric patients aged and 2 and below. Elements that reported to be helpful for caring extremes of age in the order of its importance were weekly multidisciplinary rounds, burn debriefs of selected cases, monthly performance improvement meetings/ follow ups, weekly psycho social rounds and journal clubs. Low volume high acuity patient's care was kept up to date through various interventions which were prioritized by the respondents in the order of age specific continuing education classes, professional conferences, simulation/ skills day, and journal clubs.

**Conclusions:**

The current process shows satisfactory comfort level for most of the team members to care for all age groups. Availability of pediatric and geriatric specialty professionals along with sufficient supply of age specific equipment, and ample educational opportunities were noted by the respondents as the key to providing quality care for all age groups in a single setting.

**Applicability of Research to Practice:**

The current model may be continued with sustainment of different specialty services, educational resources, and age- specific supplies and equipment.